# Parental influences on children’s dairy products consumption: a narrative review

**DOI:** 10.1017/S1368980022002555

**Published:** 2023-05

**Authors:** Ellen Greene, Celine Murrin

**Affiliations:** School of Public Health, Physiotherapy and Sports Science, University College Dublin, Belfield, Dublin 4, Ireland

**Keywords:** Children, Dairy products, Parents, Determinants

## Abstract

**Objective:**

To review research on the influence of parent-related factors on children’s dairy products consumption.

**Design:**

A search of electronic databases and a narrative synthesis of the literature were conducted. English-language articles were included if they reported data relating to parental influences on children’s consumption of dairy products and if statistical significance was reported.

**Setting:**

Studies were carried out in the USA (*n* 8) and in a range of countries across Europe (*n* 12) and Asia (*n* 5).

**Participants:**

The subjects of this research were children aged between 2 and 12 years of age, from a range of geographical locations.

**Results:**

Twenty-five studies met the inclusion criteria. The studies examined children’s dairy products consumption in relation to parental socio-economic status (education level and income) (*n* 12), home availability (*n* 2), home food environment (*n* 3), parental dairy products consumption (*n* 4), parent feeding practices (*n* 3), parents’ beliefs and attitudes (*n* 3) and parental nutrition knowledge (*n* 3). Results on the association between socio-economic status and children’s dairy products consumption varied; however, studies reporting a significant association generally observed a positive relationship. Fifteen studies reported children’s total dairy products intake as an outcome measure, with the remaining studies reporting intake of milk or other dairy products as individual foods.

**Conclusions:**

This review identified literature exploring a range of parental factors in relation to children’s dairy products intake. However, there were limited numbers of studies published within each category of modifiable factors. Further research on the parent-related determinants of dairy products consumption in children is required in order to identify potential intervention targets in this age group.

Milk and milk products are nutrient-dense foods, providing several micronutrients important for health across the lifespan, including I, K, B vitamins and Ca^(1)^. Childhood is a crucial period for skeletal growth and optimisation of peak bone mass, during which adequate Ca intake is essential^(2)^. Due to the role of Ca in bone mineralisation, inadequate intake in childhood can contribute to an increased risk of bone-related diseases, such as osteoporosis later in life^(3–5)^. Dairy products, such as milk and yogurt, are rich sources of Ca (115–120 mg/100 g of milk^(6)^) and are considered to be the most affordable source of Ca in the American diet^(7)^. Dairy products are also sources of protein, carbohydrates, unsaturated and SFA^(1)^. While excess consumption of saturated fat is of public health concern due to its association with increased risk of CVD and obesity, previous research has demonstrated a neutral or inverse association between milk and dairy products consumption and obesity and measures of adiposity in childhood^(8)^. International dietary guidelines generally recommend the consumption of two to three servings of dairy products daily for children aged under 9 years, and three to five servings are recommended for children above 9 years^(9)^. Despite recommendations and its nutritional contribution to the diet, cow’s milk consumption among children in developed countries such as Ireland, the USA, France and Germany has decreased over the past decade^(9–11)^.

In a systematic review of interventions promoting dairy products intake among school-aged children, all interventions targeting dairy products or Ca intake alone were deemed to be effective in improving dairy products consumption; however, the majority of the reviewed interventions promoted dairy products intake as part of a larger dietary intervention^(12)^. Similarly, *Srbely et al.* reported a lack of interventions targeting dairy products alone in preschool-aged children^(13)^. This highlights the need for effective dairy products interventions, which require an understanding of the determinants of dairy products consumption throughout childhood.

A wide variety of personal, interpersonal and environmental factors influence eating behaviours in children. While childhood is a vital period for physiological development, it is also a crucial life stage for the development of food behaviours and preferences. Taste preferences influence food choice throughout the lifespan and those developed early in childhood can track into adolescence and adulthood^(14)^. However, food preferences and behaviours can be modified, particularly when a variety of interpersonal and environmental influences emerge with increasing age^(15)^. There is evidence suggesting that dairy products consumption decreases with age, from childhood into adolescence^(9)^. Therefore, it is important to promote healthful eating, including dairy products consumption, throughout childhood; from the early years where lasting food preferences are developed into late childhood, where the number of exposures influencing food behaviour increases. Intervening prior to adolescence may be effective in preventing this decline in dairy products consumption and in allowing for the involvement of parents, while the direct role of parents in determining their children’s diets remains prominent.

Parents and familial environment have an important influence directly and indirectly on children’s food preferences and consumption^(16)^. As parents are often the main providers of food for children, they can directly affect the availability of healthful foods, but may also influence children’s behaviour through modelling^(16)^. The ways in which parents influence a child’s diet, which are commonly referred to in the literature, include (but are not limited to) parental knowledge, self-efficacy, attitudes and knowledge, socio-economic status and education level, motives for food choice, feeding style/parenting practices, role modelling and early life feeding, many of which may interconnect^(16–18)^. Despite this important influence, just three interventions reviewed by Hendrie *et al.* included a parental or familial component^(12)^. Additionally, Srbely *et al.* recommend the inclusion of parents in preschool dairy products interventions^(13)^. To consider parents’ inclusion in childhood dietary interventions promoting dairy products intake, the complex parental factors influencing children’s food consumption, and more specifically, dairy products consumption should be further understood.

Existing reviews on the factors affecting children’s diet examine parents’ influence on children’s overall diet quality and specifically fruit and vegetable, sugar-sweetened beverage and water consumption^(19–22)^. Within such reviews, a wide range of parent-related exposures were identified, including feeding practices, socio-demographic and home environmental factors. While similar factors were studied across each review, associations with children’s diet were unique to each food type. For example, positive parental modelling was positively associated with fruit and vegetable consumption and negatively associated with sugar-sweetened beverage consumption^(19,22)^, but there was no evidence of association with water consumption in a review conducted by Franse *et al.* ^(21)^. This highlights the need for research on the determinants of dairy products consumption in isolation, to explore the unique influences on its consumption. To our knowledge, there is a lack of literature reviewing parents’ influence on dairy products consumption specifically. The aim of this review is to explore the parent-related determinants of dairy products consumption among 2–12-year-old children.

## Methods

A narrative review of the literature was conducted. The literature search of the PubMed, Embase and psycINFO databases was conducted in October and November of 2020. This search was updated in January 2021. The search was limited to papers published in the years 2001 to 2021. The search terms used are outlined below (Table 1), relating to the population, exposure and outcome of interest.

**Table 1:** Search terms for literature search

**P**	Population	(child* OR preschool* OR pre-school* OR toddler)
**I**	Intervention (exposure)	AND (parent* AND (attitude* OR consumption OR motive* OR influence OR determinant*))
**O**	Outcome	AND (Milk OR dairy)

Additional literature was identified through Google search and searching reference lists. The first author selected the papers to be included in the review, based on the following criteria, agreed upon by the authors: Studies conducted among children (aged 2–12) which investigated the influence of parent-related factors on milk or dairy products consumption were included. Articles were included if they were published in an academic journal, in English. All types of study design were considered, provided statistical significance was reported.

### Data extraction

Extraction of key data on each study was conducted using an Excel spreadsheet. The data extracted included first author, year of publication, study design, population characteristics (sample size, age, gender, country), dairy products outcome, parent-related exposure and results. When consumption of dairy products was reported separately for each dairy product, milk, yogurt and cheese consumption alone were considered for inclusion in the review and not flavoured milks or milk-based desserts. Study quality was not examined due to the heterogeneity of the studies included.

## Results

### Overview of studies

**Fig. 1:**
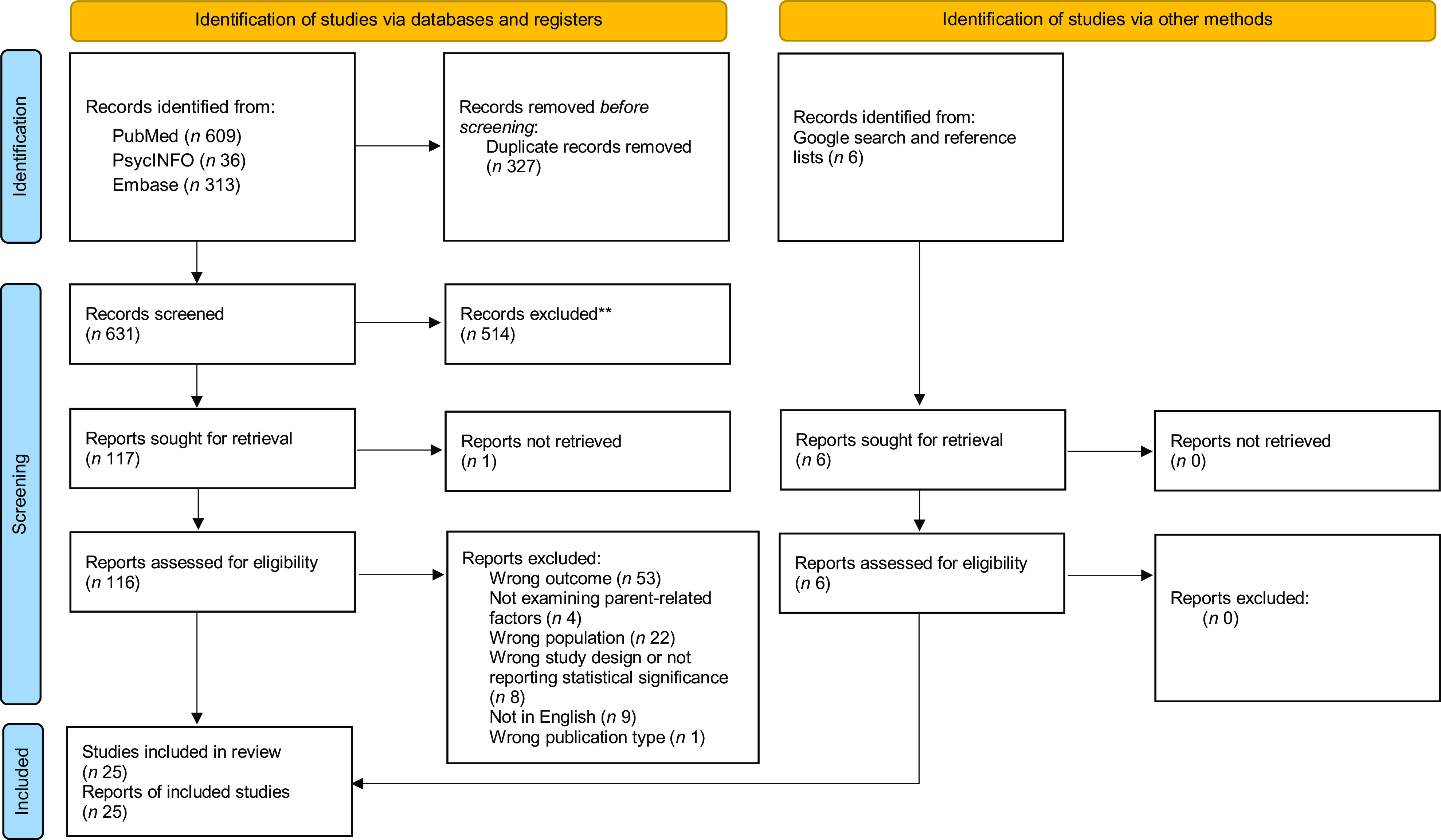
PRISMA Flow Diagram, detailing the search and manuscript screening conducted for the present review^(23)^

The search yielded 631 unique articles for abstract and title screening (Fig. 1)^(23)^, using the screening application, Rayyan^(24)^. The full texts of 117 articles were subsequently screened. Twenty-five articles were selected for final inclusion in the review^(25–49)^. The most common reasons for exclusion of full texts were lack of data looking at the influences on dairy products consumption alone and populations outside of the age criteria.

Eight studies were conducted among preschool-aged children (2–5 years), 11 were conducted among school aged-children (6–12 years) and six were conducted among children at varying stages of childhood (2–12 years). Fifteen studies examined the influence of parent-related factors on the total consumption of dairy products and 10 examined the parental influences on milk separately. Table 2 outlines the details of the reviewed studies, which will be discussed under the following headings: socio-economic status; availability, parent consumption, parent feeding practices; home food environment; beliefs and attitudes and parental knowledge.

**Table 2:** Characteristics of studies examining the factors influencing dairy products consumption

Author, year	Study Type	Aim	Population	Dairy products outcome		Parent-related exposure (s)		Results
				Food type	Method/unit of measurement	Exposure	Measure	
Asakura, 2017	Cross-sectional	To examine the relationship between nutrition knowledge and dietary intake.	Sample size: 1210 Age: 6–12 years Gender: 50·3 % male, 49·7 % female Country: Japan	Grouped: milk and dairy products	Method: Brief-type, self-administered diet history questionnaire for school children and adolescents Unit: g/4184 kJ	Parent/guardian nutrition knowledge	Percentage of correct answers to Nutrition Knowledge Questionnaire	Parent/Guardians’ nutrition knowledge was not significantly associated with children’s milk and dairy products consumption (*P* > 0·05).
Beydoun, 2009	Cross-sectional	To study the association in dietary intakes and patterns between parents (aged 20–65 years) and their children (aged 2–18 years).	Sample size: 4244 parent–child dyads Age: 2–18 years Gender: 50·3 % male, 49·6 % female Country: United States	Grouped: dairy products	Method: Two 24-h dietary recalls Unit: g/d	Parent intake dairy products (grouped)	Method: Two 24-h dietary recalls Unit: g/d	There were weak positive correlations between parent and child dairy products intake (0·1–0·3; *P* < 0·05). There were patterns of interaction with gender dyads in the intakes of dairy products (*P* < 0·05 for dyad × parental intake), where multivariate-adjusted correlations in mother–daughter dyads were significantly stronger compared to their father–child counterparts.
Bottino, 2019	Cross-sectional	To examine associations between housing instability and poor diet quality in a sample of urban parents and children.	Sample size: 340 parent-child dyads Age: 3–10 years Gender: 48·2 % male, 51·8 % female Country: United States	Grouped: dairy intake	Method: Harvard Service FFQ (HSFFQ) Unit: dietary component score (greater score indicates greater consumption)	1. Housing instability 2. Parental dairy products intake (grouped)	1. Answers to questions about housing stress factors, adapted from American Housing Survey 2. Method: HSFFQ Unit: Dietary component score	1. There was no significant association between household instability and children’s dairy products dietary component score. 2. There was a significant positive correlation between parent and child HEI score for dairy products intake. (*r* = 0·33, *P* < 0·001).
Durão, 2015	Cross-sectional	To evaluate the association between maternal perceived responsibility and child feeding practices and dietary inadequacy of 4-year-old children	Sample size: 4122 mother-child dyads Age: 4 years Gender: 51·1 % male, 48·9 % female Country: Portugal	Total dairy products (milk, yogurt and cheese combined)	Method: FFQ Unit: Adequacy intervals (adequacy defined as 3–5 times/d)	Maternal perceived responsibility and child feeding practices	Scores generated from the Child Feeding Questionnaire (CFQ) and the scales of overt and covert control	Children whose mothers reported higher levels of pressure to eat were significantly more likely to consume dairy products above the interval defined as adequate (3–5 portions/d) (OR = 1·08; 95 % CI (1·01, 1·17); *P* < 0·05). Perceived responsibility was also significantly positively associated with the odds of children consuming dairy products above this interval (OR = 1·12; 95 % CI (1·00, 1·25); *P* < 0·05).
Eloranta, 2011	Cross-sectional	To investigate nutrient intake, food consumption and meal pattern in Finnish girls and boys 6–8 years of age, and the associations of socio-economic background with these dietary factors.	Sample size: 424 Age: 6–8 years Gender: 50·2 % male, 49·8 % female Country: Finland	Skimmed milk Yogurt Cheese	Method: Four-day food records Unit: Proportion of children consuming skimmed/lower fat versions versus higher fat versions	Socio-economic status	1. Annual household income 2. Parental education	Children whose parents were in the highest income group were 2·43 times more likely to consume skimmed milk (*v*. other milk types) than in children whose parents were in the lowest income group (*P* = 0·017). Consumption of skimmed milk did not differ by parent education level (*P* > 0·05). No statistically significant associations of income or education with consumption of cheese (full fat *v*. low fat) or yogurts (full fat *v*. low fat).
Jackson, 2015	Cross-sectional	To determine if family-home nutrition factors are associated with dietary intake in rural children and to determine if food insecurity is associated with dietary intake in rural children.	Sample size: 102 Age: mean 8·4 years (elementary school up to grade 6) Gender: 46·1 % female Country: United States	Grouped: Dairy intake	Method: FFQ (The Block Kids Food Screener) Unit: Cups/d	1. Family home nutrition factors 2. Risk of food insecurity	1. Components and total score from Family Nutrition and Physical Activity screening tool 2. A yes response to at least one item on a 2-item food insecurity screening instrument	1. If parents reported that their children frequently consumed low-fat milk at meals or snacks, then dairy products intakes were significantly higher (*β* = 0·31, *P* < 0·001). There were no significant associations observed between dairy products intake and other home factors. 2. There were no significant associations between food insecurity and dietary intake.
Kano, 2020	Cross-sectional	To investigate whether a child’s food intake would differ depending on the caregiver’s perception of their child’s dietary habits	Sample size: 136 Age: 4 years Gender: 47·1 % male, 52·9 % female Country: Japan	Grouped: Dairy product intake (full-fat milk; low-fat milk; yogurt and yogurt drink; cheese; ice cream)	Method: Brief-Type Self-Administered Diet History Questionnaire Unit: g//4184 kJ/d	Parental perception of their child’s dietary habits	Parents’ perceptions of their child’s diet quality: good, bad or moderate	Dairy products intake did not significantly differ between groups of parents who perceived their child’s diet to be good, normal or poor (*P* > 0·05).
Lin, 2012	Prospective cohort study	To assess the association of dairy product consumption prospectively with adolescent obesity.	Sample size: 3679 Age: 11 years (followed up at 13 years, but cross-sectional analysis at 11 years discussed in this review) Gender: 47·8 % male, 50·6 % female Country: China	Milk consumption Non-milk dairy products	Method: Food frequency questions in questionnaire Unit: Frequency of consumption	Socio-economic status	1. Household income 2. Parental education level	There was a significant, positive association between children’s both non-milk product consumption and milk consumption with parental education and household income at 11 years (*P* < 0·001).
Lo, 2015	Cross-sectional	To evaluate the influence of parental feeding styles on children’s dietary patterns	Sample size: 4553 Age: 2–5 years Gender: 51·8 % male, 48·2 % female Region: Hong Kong	Grouped: Dairy products intake	Method: Frequency questions in questionnaire Unit: Proportion of children meeting/not meeting dairy products recommendation (2 servings or more)	Parent feeding style	Instrumental feeding, emotional feeding, prompting and encouragement to eat, control over eating (using Parental Feeding Style Questionnaire)	Adjusting for parent and child demographics, prompting and encouragement to eat was associated with 39·2 % higher likelihood of meeting dairy products requirements and control over eating was associated with 26·4 % lower likelihood of meeting dairy products requirements (*P* < 0·05).
Metcalfe, 2018	Cross-sectional	To examine the influences and correlates of involvement in family food preparation in children at ages three and four.	Sample size: 497 Age: 3 and 4 years Gender: 51·1 % male, 48·9 % female Country: United States	Milk consumption	Method: Children’s Nutrition Questionnaire Unit: Frequency of consumption/d	family food involvement	Mean score from family food involvement subscale from Comprehensive Feeding Practices Questionnaire	Milk consumption was not significantly associated with family food involvement at age 3 or 4 (*P* > 0·05)
Milla Tobarra, 2018	Cross-sectional	To explore if a relationship can be established between socio-economic status and beverage consumption in Spanish children.	Sample size: 182 Age: 9–11 years Gender: 40·6 % female Country: Spain	Skimmed or semi-skimmed milk Whole milk	Method: Young Adolescents Nutrition Assessment on Computer Unit: (ml/d)	Socio-economic status	Index calculated based on parental occupation and parental education level.	There was no significant association observed between socio-economic status and children’s consumption of whole milk (*P* = 0·370) or skimmed milk (*P* = 0·258).
Mohd Shariff, 2015	Cross-sectional	To examine energy, nutrient and food group intakes of urban children by household income status.	Sample size: 749 Age: 1–10 years Gender: 48·9 % male, 51·1 % female Country: Malaysia	Grouped: milk and dairy products	Method: 24-h recall and food record Unit: Proportion of children meeting recommendation for milk and dairy products/d (three servings for 1–3 years; two servings for 4–10 years)	Socio-economic status	Household income (low, middle, high)	Significant differences in percentages of children not meeting dairy products recommendations differed between groups of low, middle and high income among the 4–6-year-old group (*n* 252, *P* < 0·05) and the 7–10-year-old group (*n* 316, *P* < 0·05) but not for the 1–3-year-old group (*n* 181). In groups where there was a significant difference, percentage of children not meeting dairy products recommendations decreased with higher income.
Patrick, 2005	Cross-sectional	To examine how feeding styles are associated with availability, attempts to get the child to consume and child’s consumption of dairy products, fruit and vegetables in preschool children.	Sample size: 231 Age: 3–5 years Gender: 45 % male, 55 % female Country: United States	Grouped: Dairy products consumption (yogurt, cheese, milk, milk-based desserts, milk-based soups)	Method: Frequency questions in questionnaire Unit: Frequency of consumption	Feeding practices	Authoritarian *v*. authoritative style (using The caregiver’s feeding style questionnaire)	Children of caregivers who were more (relative to less) authoritative were more likely to consume dairy products (*P* < 0·001). No association was observed between dairy products consumption and an authoritarian feeding style.
Pinket, 2015	Cross-sectional	to study the quantity of water intake from beverages among preschoolers; to provide an overview of the volume and quality of beverages consumed by preschoolers; to examine the associations between water/beverage intake and gender and SES	Sample size: 7051 Age: 3·5–5·5 years Gender: 52 % boys, 48 % girls Country: Belgium, Bulgaria, Germany, Greece, Poland and Spain	Plain milk consumption	Method: FFQ Unit: ml/d	Socio-economic status	Mother’s education level (less than 14 years education or more than 14 years education)	Socio-economic status was not significantly associated with plain milk consumption in the total sample and mixed results were seen in the country specific samples. In Polish preschoolers, plain milk was significantly more consumed by preschool children from low socio-economic status (SES) backgrounds than by children from high SES backgrounds (*P* < 0·05). Belgian preschool children of lower SES mothers consumed less plain milk than those from high SES mothers (*P* < 0·05).
Raynor, 2011	Cross- sectional secondary analysis	To study the relationship between child and parent liking, and parent and child intake of fruits, vegetables, low-fat dairy products, snack foods and sweetened beverages in 4- to 9-year-old overweight/obese children.	Sample size: 135 Age: 4–9 years Gender: 63 % female, 37 % male Country: United States	1. Liking of low-fat dairy products 2. consumption of low-fat dairy products	1. Hedonic rating 2. Method: Three-day food records. Unit: Mean number of servings over 3-d period	1. Liking of low-fat dairy products 2. consumption of low-fat dairy	1. Hedonic rating 2. Mean number of servings over 3-d period	Among children with overweight and obesity, child liking and parent liking were not significantly associated with child intake of low-fat dairy products. After demographic and anthropometric variables were controlled, parent low-fat dairy products intake was positively related to child intake of low-fat dairy products (*R* ^2^Δ = 0·169; *P* < 0·001).
Robson, 2015	Cross-sectional secondary analysis	To describe the dietary intake away from the childcare centre for preschool-aged children and to examine the relationships between energy intake away from the centre with weight status, food group consumption and low-income status.	Sample size: 339 Age: 3–6 years Gender: 50 % female Country: USA	Plain milk consumption	Method: Food Record Unit: Servings/d	Socio-economic status	Low-income status measured through eligibility for Child and Adult Care Food Program	Low-income status was not significantly associated with milk consumption.
Romanos-Nanclares, 2018	Cross-sectional	To assess the association of parental nutrition knowledge and healthy-eating attitudes with their children’s adherence to the Mediterranean dietary pattern and micronutrient adequacy.	Sample size: 287 Age: 4–7 years Gender: 50·9 % male, 49·1 % female Country: Spain	Grouped: dairy products consumption	Method: Semi-quantitative FFQ Unit: g/d	1.Nutrition knowledge 2. Healthy eating attitudes	1. Score from 10 questions assessing knowledge of children’s dietary recommendations 2. Score from 8 questions assessing attitudes	Grams of dairy products consumed/d was not significantly associated with parents’ nutrition knowledge score (*P* = 0·114) or healthy eating attitudes score (*P* = 0·698).
Rongen, 2019	Cross-sectional	To investigate the content and quality of lunches consumed by Dutch primary schoolchildren aged 4 to 12 on schooldays and to examine the role of socio-economic position in the difference between lunch consumption at home and at school.	Sample size: 363 Age: 4–12 years Gender: 50·1 % male, 49·9 % female Country: The Netherlands	Cheese/cheese products milk and other dairy products	Method: Two 24-h dietary recalls. Unit: proportion of users	1. Place of consumption 2. Parent education level	1. Consumption at school and at home 2. Low middle or high level of education	There was no significant difference in cheese consumption or consumption of other dairy products consumed at home or in school (*P* < 0·05). Children of at least one parent with a high educational level consumed statistically more of the “milk and other dairy products” group than their counterparts in the lower educational level who ate lunch at school (*P* = 0·04). For those who ate lunch at home, there was no significant difference.
Roos, 2011	Cross-sectional	To examine the relationships between parental family food choice motives and children’s intakes of ‘nutrient-dense’ and ‘energy-rich foods’.	Sample size: 564 Age: 10–12 years Gender: 48·5 % male, 51·5 % female Country: Finland	Milk consumption Yogurt consumption	Method: FFQ Unit: weekly mean intake	food choice motives	Health and natural content, Mood, Sensory appeal, Price, Ethical concerns, Convenience, Weight control, Familiarity	In spearman correlation analysis, milk was not significantly associated with parents’ food choice motives (*P* > 0·05). Yogurt consumption was negatively associated with ethical concerns as a food choice motive (*P* < 0·01).
Sausenthaler, 2006	Prospective cohort study	To analyse the association between socio-economic indicators and diet among 2-year-old children, by assessing the independent contribution of parental education and equivalent income to food intake.	Sample size: 2637 Age: 2 years Gender: no data Country: Germany	Milk consumption	Method: Semi-quantitative FFQ Unit: Low intake (lowest quintile) and high intake (highest quintile)	Socio-economic status	1. Parental education (low, medium or high) 2. Net household income	Milk intake was not significantly associated with parent household income (*P* > 0·05). Children who had a low intake of milk (less than one cup/d) were more likely to have parents with lower education level than those with a high intake.
Stenhammar, 2007	Cross-sectional	To examine parents’ reported and desired frequencies (practices *v*. attitudes) of their 6-year-old children’s meals, nutritional intake and lifestyle components, as well as possible obstacles and desired support with respect to higher or lower educational backgrounds.	Sample size: 176 Age: 6 years Gender: no data Country: Sweden	Milk consumption	Method: Food frequency questions as part of questionnaire Unit: Frequency: consuming milk every day *v*. consuming milk several times/d	Parental education level	Less than 12 years’ education/12 or more years’ education	Children of parents with a college degree consumed milk more often than children of parents with lower education (*P* = 0·001).
Suggs, 2016	Cross-sectional	To describe children’s dietary behaviours in terms of adherence to the Swiss Society for Nutrition 2010 dietary guidelines.	Sample size: 568 Age: 6–12 years Gender: 49·5 % male, 50·5 % female Country: Switzerland	Grouped: Milk, probiotics and dairy products consumption	Method: 7-day food logs Unit: Adherence to Swiss Society for Nutrition guidelines for dairy products consumption (3 servings/d)	Parental education level	Primary, secondary or tertiary education	There was no significant association between children’s dairy products adherence and parental education (*P* > 0·05).
Sumonja, 2013	Cross-sectional	To determine behavioural and environmental factors that influence fruit, vegetable and dairy products consumption among Serbian schoolchildren.	Sample size: 212 Age: 8–11 years Gender: 42 % male, 58 % female Country: Serbia	Dairy products consumption	Method: 24-h recall Unit: Number of servings/d	1. Behaviour and expectations 2. Availability at home	1. Children’s normative beliefs about parents’ behaviour and expectations 2. Child-reported availability in home and school	Children who consumed less than recommended number of servings of dairy product foods more often reported they would drink more milk if their parents drank milk (*P* = 0·03). Dairy products consumption was not significantly associated with child-reported availability of milk (*P* < 0·05).
Vaitkevičiūtė, 2019	Cross-sectional	To identify the associations between the BMI of 7- and 8-year-old children, dietary behaviour and nutrition-related parenting practices.	Sample size: 3969 Age: 7–8 years Gender: 50·9 % male, 49·1 % female Country: Lithuania	Grouped: Milk, cheese and yogurt/other dairy product consumption	Method: Food frequency questions as part of questionnaire Unit: Frequency of consumption	Nutrition-related parenting practices.	Control of unhealthy food, cost of and preferences for food, taking care of family food, encouragement, pressure to eat, preparation of food, food as a reward or punishment, variety of food, liberal attitude, child’s involvement, mealtime	Nutrition-related parenting practices were not associated with children’s dairy products consumption. All statistically significant correlation coefficients were weak (all < 0·01).
Zahid, 2017	Cross-sectional	To examine the relationships between sugar-sweetened and dairy products beverage intake among children (9–12 years) and home and parental factors.	Sample size: 194 Age: 9–12 years Gender: 49·2 % male, 50·8 % female Country: USA	Dairy products beverage consumption	Method: Questions from the Harvard FFQFFQ Unit: Ounces/d	1. Availability of dairy products beverages and sugar-sweetened beverages. 2. Parent dairy products beverage intake 3. Parent dairy products/Ca knowledge 4. Parent beverage nutrition knowledge	1. Sum of responses to nine questions 2. Frequency of consumption 3. Knowledge score 4. Knowledge score	The adjusted odds of child dairy products beverage intake of 8 ounces or more/d were 1·46 times higher for each additional unit (score) of parent knowledge about sugar in beverages, 1·06 times higher for each additional unit of parent dairy products beverage intake, and 1·34 times higher for each additional level of available dairy products beverages in the home (*P* < 0·05). No other parental factors included in the model were significantly associated with child dairy products beverage intake.

### Socio-economic status

Twelve studies examined the influence of socio-economic status on children’s dairy products consumption^(27,29,30,32,35,36,38,40,42,44–46)^. Within the reviewed studies, socio-economic status was most commonly measured by parental education level or household income.

Four studies examined the association between socio-economic status and milk intake in preschool-aged children^(36,38,40,44)^. No studies reported a significant association between household income and preschool children’s milk intake^(36,40,44)^, and mixed results were observed in relation to parent education level^(38,44)^. Sausenthaler *et al.* reported that in a cohort of 2637 2-year-olds, children who had a low intake of milk (less than one cup/d) were more likely to have parents with lower education level than those with a high intake^(44)^. In a large study among preschoolers from six European countries, parent education level was not significantly associated with milk consumption overall, but significant differences were observed at a country level^(38)^. Among Belgian preschool children, those whose parents had achieved a higher level of education had higher milk intakes (*P* < 0·05). Conversely, among Polish children, those with parents with a higher level of education had lower milk intakes (*P* < 0·05).

Nine studies reported data on the influence of socio-economic status on milk consumption^(29,32,35,45)^ and dairy products consumption^(27,29,30,32,36,42,46)^ among school-aged children. Milk consumption was significantly positively associated with either parental education level or household income in two studies (*P* < 0·05)^(29,32,45)^. However, the socio-economic variable which was associated with milk consumption varied. The study conducted by Eloranta *et al.* reported that children with parents in the highest income group were more likely to consume skimmed milk, *v*. other milk types than children whose parents were in the lowest income group (*P* = 0·017)^(29)^. In the same study, there was no significant association between parental education level and type of milk consumed by children (*P* > 0·05). Three out of seven studies examining the influence of socio-economic status on school-aged children’s dairy product consumption observed a positive association with parental education and/or household income^(27,32,36,42)^. The remaining studies observed no association with measures of socio-economic status^(27,29,30,46)^.

### Home availability

Two studies reported data on the influence of the availability of dairy products on consumption, both of which were conducted among school-aged children^(47,49)^. Zahid *et al.* examined the influence of parental factors on 9–12-year-old children’s beverage consumption^(49)^. Home availability of dairy products beverages (milk and cocoa made with milk) was positively associated with dairy products beverage intake (OR = 1·34; 95 % CI (1·03, 1·73); *P* = 0·03) and negatively associated with the availability of sugar-sweetened beverages (OR = 0·74; 95 % CI (0·53, 1·05); *P* = 0·09). Conversely, Sumonja *et al.* observed that child-reported home availability of dairy products was not significantly associated with dairy products consumption adequacy in a cohort of 8–11 year-olds (*P* > 0·05)^(47)^.

### Parent consumption

Four studies examined the association between parents’ and children’s dairy products intake with that of their parents^(26,27,39,49)^. Zahid *et al.* reported that parent dairy products beverage intake specifically was associated with child dairy products beverage intake among 9–12-year-old children and their parents (OR = 1·06; 95 % CI (1·02, 1·10); *P* = 0·01)^(49)^. Similarly, Bottino *et al.* observed a moderate correlation between parent and children’s Healthy Eating Index score for dairy products (*r* = 0·33, *P* < 0·001) and Raynor *et al.* reported that parent intake of low-fat dairy products was a significant predictor of children’s low-fat dairy product intake in a regression model (*R* ^2^Δ = 0·169, *P* < 0·001).

One of the mentioned studies was carried out among a representative sample of children from the USA, where Beydoun *et al.* reported a significant, weak correlation between parent and child dairy products consumption among 2–10-year-old children^(26)^. When parent–child correlations were stratified by gender in the overall population (2–18 years, *n* 4244), correlations for dairy products consumption for mother–daughter dyads were significantly stronger than for father–child dyads. This difference in the influence of mothers and fathers is reflected in an analysis of questionnaires, completed by 8–11-year-old children^(47)^. Sumonja *et al.*, reported a borderline significant trend, where children were more likely to meet their recommended daily dairy products intake if they reported that their mothers drank milk every day (OR = 8·56; 95 % CI (0·96, 76·25); *P* = 0·05)^(47)^. However, dairy products consumption was not associated with children’s perception of their father’s milk intake. Additionally, children who did not meet dairy products recommendations were more likely to report that they would drink milk if their parents drank milk (OR = 0·14; 95 % CI (0·35, 72·16); *P* = 0·03)^(47)^.

### Parent feeding practices

Three studies reported the influence of parental feeding style on dairy products consumption among preschool children^(28,33,37)^ and one study examined its influence among school-aged children^(48)^. No association was observed in 7–8-year-old children^(48)^. Overall, among preschool children, feeding practices pertaining to a more authoritative *v*. authoritarian parental feeding style were positively associated with children’s dairy products consumption. An analysis of feeding style questionnaires completed by 231 caregivers of 3–5-year-old children reported a significant positive association between dairy products feeding attempts and children’s dairy products consumption with a more authoritative feeding style (*P* < 0·01 and *P* < 0·001, respectively)^(37)^. Adjusting for parent and child demographics, prompting and encouragement to eat was associated with 39·2 % higher likelihood of meeting dairy products requirements in a study by Lo *et al.* ^(33)^.

The feeding practice ‘control over eating’ was not associated with dairy products consumption in a study by Durão *et al.* and was associated with a lower likelihood of children consuming two or more servings of dairy products/d in the study by Lo *et al.* ^(28,33)^. Conversely, Durão *et al.* reported that the parent feeding style component ‘pressure to eat’ was associated with an increased likelihood of 4-year-old children consuming higher than the recommended servings of dairy products/d (>5 servings)^(28)^. Maternal perceived responsibility was also associated with children consuming dairy products above the recommended interval^(28)^.

### Home food environment

Three studies assessed the influence of factors related to the home food environment on children’s dairy products intake^(30,34,48)^. A study by Jackson *et al.* examined the association between dietary intake and a range of household factors among elementary school children residing in rural areas in the USA^(30)^. The family-home nutrition factors included meal patterns, eating habits, food and beverage choices, restriction and reward, family involvement, environment, screen time and routine. Overall, dairy products consumption was not significantly associated with family-related factors; however, children whose parents reported that their child drinks low-fat milk at meals or snacks had a higher dairy products intake (*P* < 0·001)^(30)^. Similarly, family involvement (involvement of children in food shopping and meal preparation) was not significantly associated with milk consumption in a cohort of children at ages 3 and 4 (*P* < 0·05) (*n* 497)^(34)^. There was no correlation between 7- and 8-year-old children’s overall dairy products consumption and mealtime environment or children’s involvement in planning and preparing meals^(48)^.

### Parent beliefs and attitudes

Three studies examined the association between children’s dairy products consumption and parents’ attitudes, as measured through nutrition attitude score, perception of children’s diet quality and food choice motives, respectively^(31,41,43)^. In a cohort of Spanish 4–7-year-old children (*n* 287), parents’ nutrition attitude score was assessed through eight yes/no questions about parents’ attitudes to a list of nutrition statements; none of which were directly related to milk, yogurt or cheese^(41)^. There was no significant association between parental nutrition attitude score and their children’s mean daily dairy products intake (*P* = 0·698)^(41)^. Similarly, Kano *et al.* did not observe a significant association between parental perceptions and children’s daily consumption of dairy products (milk, yogurt, cheese and icecream) (*P* > 0·05)^(31)^. In this study, parental perception of their child’s diet quality was measured (good, poor or average quality) and its association with children’s actual dietary consumption was examined. Roos *et al.* examined the influence of parental family food choice motives on the dietary consumption of their 10–12-year-old children^(43)^. Milk consumption was not significantly correlated with food choice motives, but there was a significant, weak negative correlation between children’s yogurt consumption and ethical concerns as a family food choice motives^(43)^.

### Parental knowledge

Three studies overall assessed the influence of parental knowledge and children’s dairy products intake. In two studies examining the influence of parents’ overall nutrition knowledge on dairy products consumption, there was no significant association observed (*P* > 0·05)^(25)^. Both studies used parents’ total score from a knowledge questionnaire to test for association with dietary factors. Parents of 6–12-year-old children completed an eighty-four-question nutrition questionnaire assessing awareness of nutrition recommendations, sources of nutrients, physiological function of nutrients and relationship between nutrition and health outcomes^(25)^. The second study assessed parents’ awareness of children’s recommendations for ten food groups, including dairy products^(41)^.

One study examined the influence of parents’ dairy products and Ca knowledge specifically on 9- to 12-year-old children’s dairy products consumption^(49)^. Parents’ knowledge of sugar in beverages was positively associated with children’s dairy products beverage intake (OR = 1·46; 95 % CI (1·06, 1·99); *P* = 0·02), whereas Ca/dairy products knowledge and general beverage nutrition knowledge were not related to children’s dairy products beverage intake (*P* > 0·05)^(49)^.

## Discussion

This review identified literature examining the influence of parental influence on the dairy products consumption of 2–12-year-old children under the sub-categories: socio-economic status, parental beliefs and knowledge, availability, home environment and parental feeding practices. It has highlighted gaps in the literature and provided direction for future study within these areas.

The influence of socio-economic status on children’s dairy products intake was examined more frequently than other variables within this review. Although the results are inconsistent, when significant associations were observed, they generally indicated that children with parents of higher socio-economic status had a higher dairy products consumption. This is consistent with previous research that reports an association between lower socio-economic status and poor diet quality^(50)^, which is a valuable consideration when designing dietary interventions. However, further research is necessary on the modifiable parental factors potentially influencing children’s dairy products intake, such as availability, attitudes, knowledge and parental consumption for the identification of potential intervention targets.

There is little published on the influence of environment and availability on children’s dairy products consumption; however, the studies reviewed suggest that home environmental context (mealtime context, children’s involvement in meal preparation and planning) may be less influential than the social influence of parents on children’s dairy products consumption. While associations in some of the reviewed studies were weak, there clearly appears to be a relationship between parents’ dairy products-related dietary behaviours and their children’s behaviour, particularly the behaviour of their mother. Parent food modelling is a potential mechanism by which this may occur. Modelling has been shown to influence children’s sugar-sweetened beverage consumption, promote adequate fruit and vegetable consumption and involve parents physically eating foods in front of their child^(19,51)^. Results from the study by Sumonja *et al.* support this idea, as it did not assess the correlation between parents’ and children’s diet directly, but demonstrated a borderline significant association between children’s dairy products consumption and their own perception of their parents’ dietary behaviour^(47)^. Additionally, children who did not meet dairy products recommendations were more likely to report that they would drink more milk if their parents also drank milk, suggesting that children in this cohort place value in their parents’ dietary behaviour. While further research is necessary on the link between parents’ and children’s dairy products consumption and its mediating factors, particularly in preschool-aged children, this research indicates that children’s dairy products consumption may be targeted through influencing parental dietary behaviour and promotion of positive food modelling.

Parents’ feeding style is a second interpersonal factor which may play a role in children’s dairy products consumption. The reviewed studies, conducted among preschool-age children, suggest that different parent feeding practices have differing effects on children’s dairy products consumption in this age group. An authoritarian feeding style incorporates practices that attempt to closely control what a child eats, whereas authoritative feeding offers the child a choice of foods and encouragement to eat, while allowing the child to make eating decisions^(37)^. The results from the studies discussed indicate that, when allowed a certain level of autonomy over eating, preschool-aged children may be more likely choose to consume dairy products, which is consistent with literature surrounding children’s overall diet quality reporting that an authoritative feeding style generally promotes the healthiest dietary habits^(52)^.

The studies discussed suggest that feeding practices pertaining to a more authoritarian feeding style could potentially have a negative impact on children’s dairy products intake^(28,33)^. The study by Durão *et al.* demonstrated that pressuring a child to eat may contribute to overconsumption of dairy products. While adequate dairy products intake is important for growth and development, overconsumption may displace other foods in the diet. This is particularly relevant at this preschool age, where children have high nutritional requirements but low capacity for food intake^(53)^. There is a need for clear public health messaging for parents around dairy products recommendations for children. Further research is necessary, particularly in school-aged children, to further assess the influence of differing parental feeding styles.

Nutrition knowledge and beliefs are known to play a role in shaping dietary behaviour; however, in this review, parental knowledge and attitudes were associated with children’s dairy products consumption in just two out of six studies^(43,49)^. It is important to note that in a majority of studies discussed in this review, dairy products consumption was not the primary outcome or was examined in conjunction with several other dietary variables. Consequently, the data collection methods reflected this. All but one study assessing the influence of parental knowledge or attitudes assessed the influence of parents’ overall nutrition knowledge or beliefs regarding general diet quality, and not dairy products-related knowledge or beliefs exclusively. One study which measured parents’ attitudes towards a list of nutrition statements did not include an item directly related to milk, yogurt or cheese on the questionnaire^(41)^. While it is valuable to assess parents’ general nutrition knowledge and healthy eating attitudes on their child’s dairy products consumption, it is necessary to further explore perceptions around dairy products specifically, in order to identify potential targets for dairy products interventions and to truly understand if attitudes and knowledge influence children’s dairy products intake.

Overall, this review identifies a need for more specificity when examining the influences on children’s dairy products consumption. Fifteen studies measured dairy products intake as a total score or pooled intake of several dairy products, many of which do not clearly state which dairy products are incorporated in this total. In three studies, this outcome measure included products such as milk-based desserts and flavoured milk^(31,37,48)^, consumption of which is not recommended at the same level as the consumption of milk, yogurt or cheese. While these products are categorised under the same food group, they differ in taste, culinary use and nutritional value in terms of sugar and fat content. Therefore, examining the influences on milk, yogurt and cheese intake individually may be more appropriate. Furthermore, as children’s milk consumption is decreasing^(9)^, further research should be carried out on the determinants of its consumption alone. Ten studies within this review considered milk consumption individually, six of which examined milk consumption according to measures of socio-economic status only. This identifies a gap in the literature looking at the influence of modifiable factors on children’s milk consumption.

### Limitations

A limitation of this literature review is the age limit set, as part of the inclusion criteria. While research conducted among adolescents was beyond the scope of this review, the age cut-off of 12 years resulted in the loss of potentially valuable data in cases where children aged older than 13 or older were included within a child sample, and data were not presented separately.

The subject area examined is broad and the reviewed studies assessed a range of parental factors and their influence on children’s dairy products consumption, which were categorised under several headings. However, there is little published within each category. There is also heterogeneity within some categories discussed, particularly in how parental attitudes and knowledge were measured. Therefore, conclusions cannot be drawn based on the limited number of studies within each category and heterogenous nature in which outcomes and exposures were measured.

Study design and methodological aspects of the studies reviewed posed several limitations. The lack of consistency in the measurement and reporting dairy products consumption does not allow for direct comparison between studies. Some studies reported average consumption of milk and individual dairy products, while others reported grouped consumption of dairy products or dairy consumption scores. As fifteen studies reported children’s total dairy products consumption, it is difficult to determine the influences on individual types of dairy products. Furthermore, most studies reviewed were cross-sectional in design, lacking the ability to demonstrate the temporal nature of parental influences.

Previous literature has shown that parental influence on children’s general diet quality can differ between parents’ gender^(16)^. Two of the studies reviewed suggested a difference in the influence of mother’s and father’s consumption on children’s dairy consumption^(26,47)^. However, all but one study reviewed, which reported the gender of responding parents, had parental populations primarily comprised of mothers or female caregivers. Inclusion of fathers or male caregivers in future analyses is necessary to further explore their influence on children’s dairy products consumption and to identify differences between the influence of mothers and fathers.

## Conclusion

A range of parental factors have been discussed in relation to children’s dairy products consumption; however, little is published within each category of parental influence. Further research is needed on the influence of modifiable parental factors, particularly on children’s milk consumption specifically, to identify potential intervention targets. Due to the limited number of studies conducted on modifiable parental factors and the heterogeneity of the studies reviewed, it is difficult to draw conclusions. However, it appears from the limited number of studies available that social influences such as parent feeding practices and parent consumption of dairy products, may have an influence on children’s dairy products consumption. Further research is necessary on the influence of parents on children’s consumption of individual dairy products and on the interaction between these factors in influencing children’s dairy products consumption.
